# Supplementary material for: The interactome of intact mitochondria by cross-linking mass spectrometry provides evidence for coexisting respiratory supercomplexes
Source: Mol Cell Proteomics. 2017 Dec 8;17(2):216–32. doi: 10.1074/mcp.RA117.000470 (PMC5795388; doi:10.1074/mcp.RA117.000470)
Supplement: Supplemental Data [file supp_RA117.000470_133922_0_supp_23978_fzffwf.zip › spectra_annotation/mito_spectra_annotation/349-1-6-1-6-1.pdf]

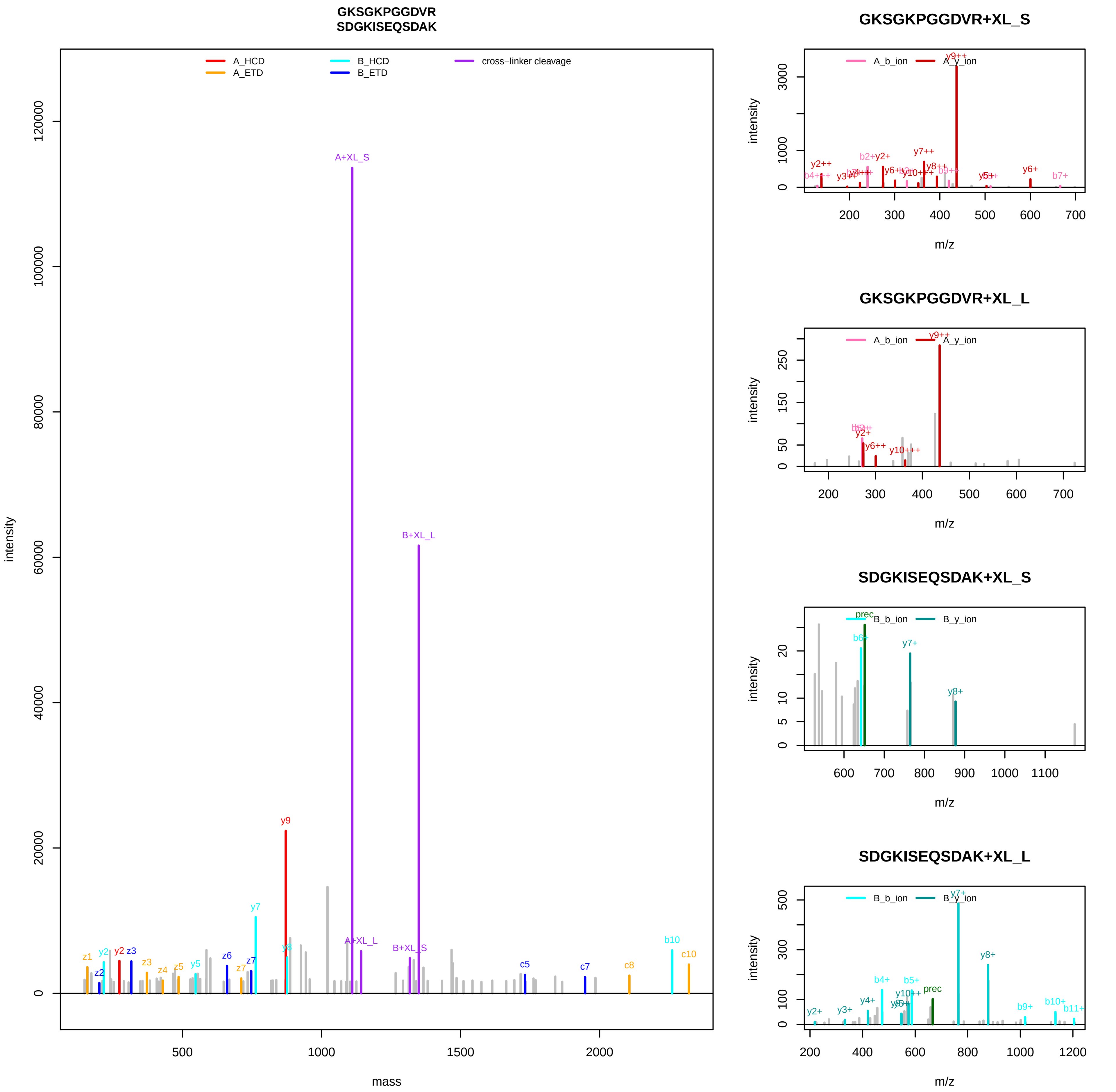
GKSGKPGGDVR
SDGKISEQSDAK
A_HCD
A_ETD
B_HCD
B_ETD
cross−linker cleavage
A+XL_S
B+XL_L
A+XL_L
B+XL_S
y9
y7
b10
c10
c8
c7
c5
z1
y2
z2
z3
z4
z5
y5
z6
z7
y8
intensity
mass
0
20000
40000
60000
80000
100000
120000
500
1000
1500
2000
GKSGKPGGDVR+XL_S
A_b_ion
A_y_ion
y9++
y7++
y6+
b7+
m/z
200
300
400
500
600
700
0
1000
3000
GKSGKPGGDVR+XL_L
y2+
y6++
0
50
150
250
SDGKISEQSDAK+XL_S
B_b_ion
B_y_ion
prec
y7+
y8+
0
5
10
20
600
700
800
900
1000
1100
SDGKISEQSDAK+XL_L
y7+
y8+
b4+
b5+
y4+
y3+
y2+
b9+
b10+
b11+
0
100
300
500
200
400
600
800
1000
1200
